# New insights for C5a and C5a receptors in sepsis

**DOI:** 10.3389/fimmu.2012.00368

**Published:** 2012-12-10

**Authors:** Chunguang Yan, Hongwei Gao

**Affiliations:** Department of Anesthesiology, Perioperative and Pain Medicine, Center for Experimental Therapeutics and Reperfusion Injury, Brigham and Women's Hospital, Harvard Medical School, Harvard Institutes of MedicineBoston, MA, USA

**Keywords:** sepsis, C5a, receptor, inflammation, complement

## Abstract

The complement system plays a central role in inflammation and immunity. Among the complement activation products, C5a is one of the most potent inflammatory peptides with a broad spectrum of functions. There is strong evidence for complement activation including elevated plasma level of C5a in humans and animals with sepsis. C5a exerts its effects through the C5a receptors. Of the two receptors that bind C5a, the C5aR (CD88) is known to mediate signaling activity, whereas the function of another C5a binding receptor, C5L2, remains largely unknown. Here, we review the critical role of C5a in sepsis and summarize evidence indicating that both C5aR and C5L2 act as regulating receptors for C5a during sepsis.

## Introduction

**Figure 1:**
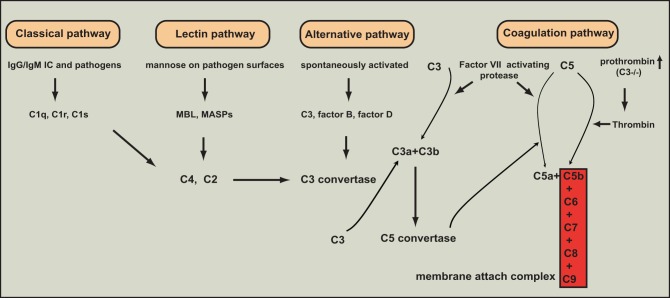
**The various pathways of complement activation.** Four major pathways are involved in complement activation: classical, lectin, alternative, and coagulation pathways.

The complement system is composed of more than 30 heat-labile plasma proteins (Guo and Ward, 2005). Although complement activation plays a key role in innate immune defenses against invading bacteria, over-activation of complements leads to many inflammatory diseases including sepsis (Huber-Lang et al., 2001a,b, 2002a,b; Laudes et al., 2002b; Guo et al., 2004; Guo and Ward, 2005; Rittirsch et al., 2008). The complement system acts as an enzymatic cascade through a variety of protein-protein interactions, and complement activation occurs after a variety of different stimuli. Three well-known pathways are involved in complement activation: classical pathway, mannose-binding lectin (MBL) pathway, and alternative pathway (Guo et al., 2004; Guo and Ward, 2005) (Figure 1). The classical pathway can be activated by direct association of C1q with the microbial pathogen surfaces. It can also be initiated by binding of C1q to antigen-antibody complexes during an adaptive immune response. The MBL pathway is trigged by binding of MBL to carbohydrate structures containing mannose present on bacterium or virus surfaces. The alternative pathway is activated by binding of spontaneously activated complement C3 protein (C3b fragment) to pathogen surfaces. All the three pathways result in a series of enzymatic cleavage reactions, leading to formation of C3 convertase, at which the three pathways converge (Guo and Ward, 2005). C3 convertase can lead to the formation of C3a, C3b, C5a, C5b, C6, C7, C8, and C9, among which C5b, C6, C7, C8, and C9 form a membrane attack complex (C5b-9), which is used by host to lyse gram-negative bacteria. Coagulation pathway was recently suggested as a novel pathway of complement activation acting-independently of the formation of canonical C3/C5 convertases (Huber-Lang et al., 2006) (Figure 1). In this pathway, thrombin functions as a C5 convertase in the absence of C3, leading to the production of C5a and formation of C5b-9 (Huber-Lang et al., 2006). Moreover, in multiple trauma patients, factor VII-activating protease (FASP), which was activated by circulating nucleosomes released from necrotic cells, interacted with complement proteins in plasma, and cleaved C3 and C5 to produce C3a and C5a (Kanse et al., 2012). However, the mechanistic basis underlying the interaction between coagulation pathway and complement pathway remains poorly understood.

Sepsis represents a spectrum of clinical symptoms characterized by the inability of host to regulate the inflammatory response (Riedemann et al., 2003b). In the United States, it affects at least 600,000 persons per year, leading to around 250,000 annual deaths (Ward, 2010; Bosmann et al., 2011b). The systemic inflammatory response syndrome (SIRS), sepsis, severe sepsis, septic shock, and multiorgan failure (MOF) are currently used to characterize the progressive stages of this very complex and therapeutically challenging disorder of the immune and inflammatory systems (Hoesel et al., 2006). Bacterial infections can progress to sepsis, but detection of bacteremia is not a prerequisite for making the clinical diagnosis of sepsis. Sepsis can stimulate complement activation in both humans and animals, resulting in increased levels of C3a, C4a, and C5a in plasma (Bengtson and Heideman, 1988; Smedegard et al., 1989; de Boer et al., 1993; Nakae et al., 1994). It has been demonstrated that classical, MBL, and alternative pathways all participate in complement system activation, and play important roles in sepsis (Celik et al., 2001; Windbichler et al., 2004; Dahlke et al., 2011). Importantly, a recent study using CLP-induced sepsis model in mice lacking either the alternative (fD^−/−^) or classical (C1q^−/−^) complement activation pathway provides clear evidence that the classical pathway and the alternative pathway exert distinctly different contribution to the innate host response during sepsis by showing that the classical pathway is important for clearing bacteria in the early development of sepsis, whereas the alternative pathway may play a more important role for the later phase of development (Dahlke et al., 2011). During sepsis, over-activation of complement system causes multiple organ damage and compromised immune responses (Guo et al., 2003). Among complement system, C5a is the most powerful inflammatory mediator, which can lead to adverse systemic consequences by a broad spectrum of mechanisms in sepsis (Ward, 2004; Guo and Ward, 2005). C5a exerts its effect through its receptors: C5aR and C5L2. The roles of C5a signaling in inflammatory injury associated with sepsis are becoming defined. Here, we review the recent data for the critical roles of C5a, C5aR, and C5L2 during sepsis.

## Role of C5a in sepsis

Human C5a is composed of 74 amino acids, which is a glycosylated peptide. NMR spectroscopy demonstrated that C5a contains four helices, which are connected by loops. The helical structures are cross-linked by disulfide bonds, which make the molecule quite stable in the presence of oxidative stress (Ward, 2010). It has been well established that C5a production could be due to plasma complement activation pathways. In addition, studies indicated that C5a could also be generated through cleavage of C5 by phagocytic cell-derived serine proteases that have C5 convertase activity (Huber-Lang et al., 2002c). These cells include alveolar macrophages and neutrophils (Huber-Lang et al., 2002c). Interestingly, a recent study shows that M-ficolin, a pattern-recognition molecule which activates the complement system in a manner similar to MBL pathway, was released by phagocytes during bacterial sepsis, and its cord blood level was positively related to circulating phagocytes and early-onset sepsis in neonates (Schlapbach et al., 2012).

The roles of C5a in sepsis have been investigated in subhuman primate model of sepsis-induced by intravenous injection of *Escherichia coli* (*E. coli*) into monkeys. In this model, C5a neutralizing antibody reduced several septic parameters (Stevens et al., 1986; Hangen et al., 1987). As a result, all septic animals treated with anti-C5a antibody survived, and did not developed severe lung edema and decreased oxygenation (Stevens et al., 1986; Hangen et al., 1987). In contrast, 75% of animals treated with control IgG died with decreased oxygenation, increased extravascular lung water, and profound hypotension (Stevens et al., 1986; Hangen et al., 1987).

The molecular mechanisms underlying the harmful effects of excessive C5a on innate immune functions during sepsis are being defined. C5a inhibited phagocytic activity of normal blood neutrophil in a dose-dependent manner (Huber-Lang et al., 2002b). Furthermore, blood neutrophils from septic rats showed defect in phagocytosis (Huber-Lang et al., 2002b). In contrast, neutrophils from cecal ligation and puncture (CLP) rats treated with antibody to C5a preserved the phagocytic activity. C5a treatments also led to suppression of p47^*phox*^ phosphorylation, and its subsequent translocation to the cell membrane and assemble of NADPH oxidase, which resulted in inhibition of respiratory burst in neutrophils (Huber-Lang et al., 2002b). C5a-induced defects in phagocytosis and NADPH oxidase assembly caused defective bactericidal activity of neutrophils, leading to increased bacterial counts (Huber-Lang et al., 2002b). In CLP-induced sepsis model, 50% of rats receiving anti-C5a antibody treatment survived during a 10-day survival study, while the survival rate was only 9.5% in the septic group treated with normal IgG (Czermak et al., 1999). The improved survival was linked to reduced bacterial colony forming-units (CFU) in blood, spleen, and liver, and improved H_2_O_2_-generating ability of neutrophils by C5a blockade (Czermak et al., 1999).

Complement activation occurs during sepsis in human, leading to the generation of anaphylatoxins including C3a, C4a, and C5a (Nakae et al., 1996). Appearance of high levels of anaphylatoxins was correlated with MOF that is a key factor resulting in death, and lower anaphylatoxin levels could only be identified in surviving septic patients but not non-surviving persons (Bengtson and Heideman, 1986; Nakae et al., 1996). In addition, *in vitro* experiment demonstrated that neutrophils in patients surviving from sepsis-induced MOF had defect in chemotactic response to C5a, which might be related with inability of C5a to bind to neutrophils (Solomkin et al., 1981; Goya et al., 1994). In experimental sepsis, C5a blockade attenuated the parameters of MOF, and maintained normal chemotactic function of neutrophils (Huber-Lang et al., 2001a; Flierl et al., 2006). Importantly, C5a blockade given at 12 h after the initiation of sepsis has protective effects against detrimental influence of septic shock (Huber-Lang et al., 2001b). However, it remains to be determined whether, in human beings with sepsis, there may be a similar “time window” during which anti-C5a treatment can be an effective method to improve survival.

## C5a regulation of inflammatory mediators

C5a promotes proinflammatory mediators' production in many cell types (Table 1). For example, C5a stimulated the synthesis and release of cytokines such as TNF-α, IL-1β, and IL-6 by human peripheral blood mononuclear cells (Schindler et al., 1990; Scholz et al., 1990). In addition, C5a promoted generation of IL-8, IL-1β, and RANTES at mRNA level in human umbilical cord endothelial cells (HUVEC) (Monsinjon et al., 2003). A recent study found that IL-17F production in mouse peritoneal macrophages was significantly induced by LPS at both mRNA and protein levels (Bosmann et al., 2011a). Interestingly, C5a amplified LPS-stimulated IL-17F generation by enhancing Akt phosphorlation in a MyD88-dependent manner (Bosmann et al., 2011a). C5a can also exert *in vivo* immunoregulatory functions (Table 2). For example, plasma level of IL-17F was dramatically elevated in both LPS- and CLP-induced septic mice, which correlated with C5a concentration (Bosmann et al., 2011a). Furthermore, IL-17F level was greatly decreased in septic mice receiving C5a blocking antibody, suggesting that IL-17F production was positively regulated by C5a during sepsis. C5a can also synergistically induce the production of cytokines and chemokines with LPS in various cells. These include IL-1 and TNF from mouse peritoneal macrophages and human monocytes (Cavaillon et al., 1990), IL-8 from human neutrophils (Strieter et al., 1992), and TNF-α, macrophage inflammatory protein-2 (MIP-2), cytokine-induced neutrophil chemoattractant-1 (CINC), and IL-1β from rat alveolar epithelial cells (Riedemann et al., 2002c). Similarly, exposure of mouse dermal microvascular endothelial cells to LPS or IL-6, followed by exposure to C5a, resulted in a synergistic effect on the generation of MIP-2 and monocyte chemoattractant protein-1 (MCP-1) (Laudes et al., 2002a). Our recent study demonstrated that C5a increased IgG immune complex-stimulated TNF-α, MIP-2, and MIP-1α expression by enhancing phosphorylation of both p38 and p44/42 MAPKs in a Fcγ receptor-dependent manner (Yan et al., 2012). C5a also plays a pivotal role in lymphocyte inflammatory responses. For example, C5a modulated IL-22 and IL-17 expressions by human CD4+ T cells (Gerard et al., 2005). Moreover, C5a-induced a robust Th1 polarization, while inhibited Th2 response in trinitrobenzene sulfonic acid-induced model of colitis, which contributed to the exacerbation of intestinal damage (Chen et al., 2011). The role of C5a in innate lymphocyte activation during *E. coli*-induced sepsis was recently reported (Fusakio et al., 2011). In this study, using C5aR^+^/C5aR^−^ mixed bone marrow chimeras, the cognate C5a/C5aR interaction on NKT cells was identified as a critical factor for NKT cell activation and the recruitment during sepsis. Furthermore, there is a synergistic interaction between C5a/C5aR and TLRs, which enhances the production of TNF-α and IFN-γ from NKT and NK cells in co-cultures with dendritic cells (DC) (Fusakio et al., 2011). DC are bridges linking innate and adaptive immunity, their functions are affected by C5a. When cultured with *Mycobacterium bovis* Bacillus Calmette-Guerin (BCG), DCs from C5-deficient mice secreted much less IL-12 in comparison with those from C5-sufficient animals (Moulton et al., 2007). Furthermore, C5-deficient DCs fully restored the IL-12 generating capacity when incubated with BCG in presence of C5a (Moulton et al., 2007), suggesting that C5a may contribute to the generation of acquired immune responses in mice by modulating Th1 response.

**Table 1:** **C5a regulation of inflammatory mediators**.

**Cell type**	**Clinical condition associated with *in vitro* model of choice**	**Stimulus**	**Signaling effectors involved**	**Immune reactions**	**References**
Neutrophil	Sepis	C5a		Reduced phagocytic activity, respiratory burst, bactericidal activity, and chemotactic response	Solomkin et al., 1981; Goya et al., 1994; Huber-Lang et al., 2002b
			PI3-K, Akt, ERK1/2, PKC, and Bcl-XL	Reduced apoptosis	Perianayagam et al., 2002; Suvorova et al., 2008
				Increased IL-6 production	Riedemann et al., 2004
		C5a + LPS	IκBα	Reduced TNF-alpha production	Riedemann et al., 2003a
			p38 and p44/42 MAPKs	Increased IL-6 production	Riedemann et al., 2004
			PI3-K	Increased IL-8 and IL-1beta production	Wrann et al., 2007
		C5a + LPS		Increased IL-6 generation in the presence of anti-C5L2 antibody treatment	Scola et al., 2009
Monocyte	Sepis	C5a		Increased TNF-alpha, IL-1beta, and IL-6 production	Schindler et al., 1990; Scholz et al., 1990
		C5a + LPS		Elevated IL-1, TNF, and IL-8 generation	Cavaillon et al., 1990; Strieter et al., 1992
		C5a + LPS/IFN-gamma		Reduced IL-12 expression	Wittmann et al., 1999
Human umbilical cord endothelial cell	Sepis	C5a		Increased IL-8, IL-1beta, RANTES, and tissue factor expressions Reduced IL-6	Ikeda et al., 1997; Monsinjon et al., 2003
Macrophage	Sepis	C5a + LPS	MyD88 and Akt	Enhanced IL-17F generation	Bosmann et al., 2011a
				Enhanced IL-1, and TNF generation	Cavaillon et al., 1990
			PI3-K, Akt, MEK1/2 and ERK1/2	Reduced IL-17A, and IL-23 expressions that are C5aR-but not C5L2-dependent while increased IL-10 production	Bosmann et al., 2012
	IgG immune complex-associated diseases	C5a + IgG IC	p38 and p44/42 MAPKs	Amplified expressions of MIP-2, MIP-1alpha, TNF-alpha	Yan et al., 2012
Macrophage lack of C5	Tuberculosis	Mycobacterium tuberculosis (MTB)		Enhanced growth of MTB Reduced secretion of TNF-alpha, IL-1beta, IL-6, IL-12, KC, MIP-2, and MIP-1alpha	Jagannath et al., 2000
Alveolar epithelial cell		C5a + LPS		Increased expressions of TNF-alpha, MIP-2, CINC, and IL-1beta	Riedemann et al., 2002c
Dermal microvascular endothelial cells	Sepis	C5a + LPS/IL-6		Increased MIP-2, and MCP-1production	Laudes et al., 2002a
CD4 + T cell	Age-related macular degeneration	C5a		Enhanced IL-22, and IL-17 generation	Gerard et al., 2005
NKT and NK	Sepis	C5a + ligands for TLRs		Increased expressions of TNF-alpha, and IFN-gamma	Fusakio et al., 2011
C5 deficient dendritic cell	Tuberculosis	*Mycobacterium bovis* Bacillus Calmette-Guerin (BCG)		Reduced IL-12 expression	Moulton et al., 2007
Adrenal medulla cell	Sepis	C5a	Caspase	Increased apoptosis	Flierl et al., 2008a
γ^d^T cell	Sepis	C5a		Enhanced C5aR, and IL-17 expressions	Haviland et al., 1995
Thymocytes from septic rats	Sepis	C5a	Caspase-3, -6, 9, cytochrome c, and Bcl-X L	Enhanced apoptosis	Guo et al., 2000

**Table 2:** ***In vivo* immunoregulatory properties of the C5a/C5aR system**.

**Model**	**Treatment**	**Outcomes**	**References**
*E. coli*-induced sepsis	C5a neutralizing antibody	Increased survival rate, decreased lung edema and oxygenation	Stevens et al., 1986; Hangen et al., 1987
	anti-C5a antibody	Decreased IL-6 level in serum	Hopken et al., 1996
	C5aR knockout	Attenuation of NK and NKT cell activation	Fusakio et al., 2011
		Reduced TNF-alpha and IFN-gamma release by NK and NKT cells	
		Impaired recruitment of NK and NKT cells to the site of infection	
		Increased survival rate	
LPS-induced endotoxic shock	C5a neutralizing antibody	Attenuated septic parameters	Smedegard et al., 1989
	C5aR knockout	Increased circulating IL-23 and IL-17A level	Van Epps et al., 1990; Bosmann et al., 2012
		Increased resistance to endotoxic shock	
	C5L2 knockout	Increased serum IL-1beta while decreased survival rate	Han et al., 2011
CLP-induced sepsis	anti-C5a antibody	Reduced bacterial colony forming-units while improved respiratory burst	Czermak et al., 1999; Laudes et al., 2002b; Guo et al., 2003, 2006; Flierl et al., 2008a, 2009; Atefi et al., 2011; Bosmann et al., 2011a
		Reduced IL-17F level in serum	
		Attenuated coagulant paremeters	
		Reduced apoptosis of adrenal medulla cell	
		Ameliorated septic encephalopathy	
		Restoration of neutrophil to spontaneous apoptosis Reduced inflammatory mediators' production by cardiomyocytes while attenuation of cardiac dysfunction	
		Restoration of C5aR content on neutrophils	
	C5aR knockout	Decreased plasma levels of IL-1beta, IL-6, MIP-2, and MIP-1alpha while increased survival rate	Rittirsch et al., 2008
	C5aR antagonist	Improved survival	Huber-Lang et al., 2002a
	C5aR antibody	Reduced IL-6 and TNF-alpha production in serum, and bacterial burden while improved survival	Zahedi et al., 2000
	anti-C5L2 antibody	Increased serum IL-6 level	
	C5L2 knockout	Decreased serum levels of IL-1beta, MIP-2, MIP-1alpha, and HMGB1 while improved survival	Rittirsch et al., 2008; Atefi et al., 2011
		Increased pro-inflammatory mediators' production from cardiomyocytes	
House dust mite-induced allergic asthma	C5/C5aR knockout	Increased IL-23 production by dendritic cells and Th17 cell differentiation and proliferation	Lajoie et al., 2010
		Enhanced airway hyperresponsiveness	
	C5L2 knockout	Attenuated asthmatic phenotye	Johswich et al., 2006
IgG IC-induced acute lung injury	C5L2 knockout	Reduced lung inflammation	Gerard et al., 2005

On the other hand, C5a can also limit the pro-inflammatory mediators' production. For example, in an experimental allergic model, C5a suppressed DC-derived IL-23 production, which led to inhibition of Th17 cell differentiation and proliferation, and limited the severe airway hyper-responsiveness (Lajoie et al., 2010). C5a can also suppress many other pro-inflammatory mediators' expression. For example, Mycobacterium tuberculosis (MTB)-infected macrophages from C5-deficient mice showed enhanced growth of MTB coinciding with a reduced secretion of both cytokines (TNF-α, IL-1β, IL-6, and IL-12) and chemokines (KC, MIP-2, and MIP-1α) (Jagannath et al., 2000). Both LPS and IFN-γ-induced IL-12 expression were markedly suppressed by C5a in human monocytes (Wittmann et al., 1999). IL-6 expression was significantly reduced by C5a in HUVECs (Monsinjon et al., 2003). Moreover, C5a significantly suppressed LPS-induced TNF-α expression by increasing the expression of cytosolic IκBα, an inhibitor of NF-κB activation, in neutrophils (Riedemann et al., 2003a). Interestingly, a recent study showed that C5a exhibited anti-inflammatory effect during endotoxic shock by suppressing IL-17A and IL-23 production from CD11b(+)F4/80(+) macrophages (Bosmann et al., 2012). Mechanistically, endotoxin-induced generation of C5a resulted in activation of the PI3-K-Akt and MEK1/2-ERK1/2 pathways, leading to IL-10 production, followed by suppression of IL-17A and IL-23 expressions (Bosmann et al., 2012).

Complement system is activated at early time during sepsis, causing C5a production, which may play a central role in generation of “inflammatory cytokine storm.” During sepsis, there is an increase of both pro-inflammatory mediators in blood including IL-6, TNF-α, IL-1β, IL-8, and IFN-γ, and anti-inflammatory factors such as IL-10, IL-13, IL-4, and TGF-β (Wolkow, 1998; Titheradge, 1999; Le Tulzo et al., 2002; Guo et al., 2004; Flierl et al., 2008b). Sepsis-induced imbalance between pro-inflammatory and anti-inflammatory responses leads to apoptosis, immunosuppression, and multiple organ dysfunction (Guo et al., 2004). Neutrophils are generally regarded as driving force for acute inflammation. The role of C5a in sepsis is best studied by its effects on neutrophil inflammatory responses. For example, a recent study demonstrated that elevated serum IL-6 level during CLP-induced sepsis was due to increased level of C5a (Riedemann et al., 2004). Importantly, neutrophil depletion resulted in a more than 50% decrease of IL-6 level, suggesting that neutrophils are the major contributor of C5a-regulated IL-6 production during sepsis (Riedemann et al., 2004). In another study, anti-C5a monoclonal antibody led to an over 75% decrease in serum IL-6 bioactivity in septic pigs receiving intravenous injection of *E. coli* when compared with control group (Hopken et al., 1996). *In vitro*, either LPS or C5a significantly induced IL-6 expression in neutrophils (Riedemann et al., 2004). Importantly, C5a enhanced LPS-stimulated IL-6 generation by rapidly inducing phosphorylation of p38 and p44/42 MAPKs (Riedemann et al., 2004). In human neutrophils, C5a significantly boosted TLR-4-dependent generation of IL-1β and IL-8, which was controlled in an inhibitory fashion by the PI3K pathway (Wrann et al., 2007). Furthermore, PI3K signaling pathway exerts an overall protective role during the onset of sepsis in rodents by limiting C5a-mediated effects on neutrophil cytokine generation, and promoting oxidative burst and phagocytosis (Wrann et al., 2007). Thus, these studies suggest a leading role of C5a in the imbalance of inflammatory network during sepsis. However, whether other cell populations such as monocytes and NKT cells are responsible for the cytokine storm *in vivo* during sepsis and how the complex interactions between these cells contribute to the acute inflammatory processes in sepsis remains a puzzle.

## C5a regulation of coagulation pathways during sepsis

During sepsis, blood monocytes, tissue macrophage, and endothelial cells serve as sensors of invading microorganisms by using pattern recognition receptors. The interactions between the host receptors and the conserved structures of pathogens lead to activation of inflammatory and coagulation pathways. It is well known that coagulation cascade is activated in septic patients. There are two pathways involved in blood coagulation: extrinsic and intrinsic pathways. Extrinsic pathway is responsible for initiation of blood clotting, and intrinsic pathway is the initiator of blood coagulation amplification (Aird, 2003). During sepsis, elevated expression of tissue factor (TF) was found on the surfaces of tissue macrophages and circulating monocytes, which led to initiation of extrinsic clotting cascade, thrombin production, and fibrin formation (Aird, 2003). At the same time, sepsis suppresses natural anti-coagulant responses, which results in increased thrombin production, fibrin formation and consumption of clotting factors, and decreased protein C in blood (Aird, 2003). Injection of exogenous protein C inhibited initiation of coagulation pathway, reduced organ dysfunction, and improved survival rate in a sepsis model performed in baboon, while *in vivo* blockade of protein C activation by using anti-protein C antibody worsened *E. coli*-induced septic shock (Taylor et al., 1987). However, due to risk of serious bleeding in 35% patients receiving rhAPC (recombinant human activated protein C), the FDA and European Medicines Agency (EMEA) have recently withdrawn their support and recommends not using the product, and the manufacturer has withdrawn the product from the market (Kylat and Ohlsson, 2012).

A number of evidences indicate the involvement of C5a in coagulation pathway. The recombinant human C5a stimulated TF expression in a dose-dependent fashion in HUVECs (Ikeda et al., 1997). In addition, C5a-induced TF production in human leukocytes (Muhlfelder et al., 1979). In the CLP-induced sepsis model, C5a neutralizing antibody ameliorated coagulation/fibrinolytic protein changes in rats, thus preventing dissemination of intravascular coagulation (Laudes et al., 2002b). In septic rats receiving anti-C5a antibody, coagulant parameters were greatly attenuated (Laudes et al., 2002b). Additionally, C5a markedly induced IL-8 generation in HUVECs (Monsinjon et al., 2003), which could in turn induce the fibrin deposition and promote thrombogenesis as well as proliferation and structural reorganization of endothelial cell (Guo et al., 2004). Therefore, the involvement of C5a in activation of coagulation pathways seems to be mediated by up-regulated expression of IL-8 in human beings, and C5a neutralizing antibody treatment may be an effective approach to prevent coagulation-induced organ damage during sepsis. The coagulation system also has profound effects on the complement activation. It has been shown that thrombin is capable of generating C5a in the absence of C3 (Huber-Lang et al., 2006). A most recent study provided novel insights into the complex interaction between the coagulation/fibrinolysis cascades and the complement system *in vitro* and *ex vivo* (Amara et al., 2010). This study established multiple links between various factors of the coagulation and fibrinolysis cascades and the central complement components C3 and C5 by demonstrating that thrombin, human coagulation factors (F) XIa, Xa, and IXa, and plasmin were all found to effectively cleave C3 and C5 (Amara et al., 2010). Thus, it is possible that C5a pathway and coagulation/fibrinolysis cascades during sepsis can regulate each other by positive-feedback mechanisms.

## Role of C5a in cell apoptosis during sepsis

**Figure 2:**
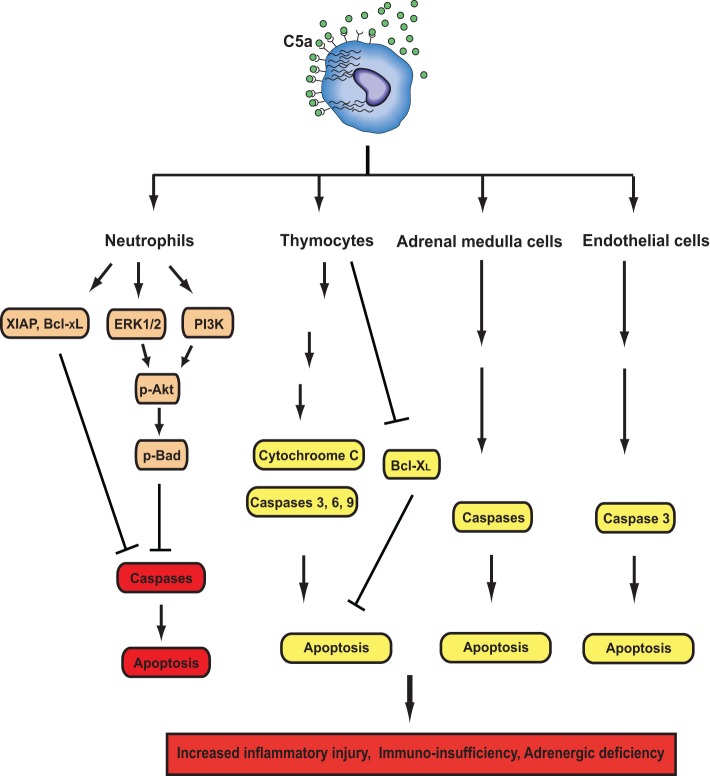
**Effects of C5a signals on apoptosis during sepsis.** C5a have both anti- and pro-apoptotic activities depending on cell types. In neutrophils, C5a activates PI3K and ERK1/2 pathways, leading to phosphorylation of Akt and subsequent phosphorylation of Bad. Phosphorylated Bad inhibits cytochrome C release from mitochondria to prevent the formation of the apoptosome, thereby inhibiting neutrophil apoptosis. C5a together with LPS induces XIAP production, which can inhibit the formation of the apoptosome. Sepsis enhances Bcl-xL expression and reduces Bim expression. C5a and LPS can also enhance Bcl-xL expression. All of these events are in favor of maintaining the integrity of mitochondria and preventing neutrophil apoptosis. In thymocyes, adrenal medulla cells, and endothelial cells, C5a can induce apoptosis by enhancing caspase activities or inhibiting Bcl-xL expression.

Immunosuppression occurs in humans and rodents during sepsis, which is due to reduced number of T and B lymphocytes in lymphoid tissues and in circulation (Guo et al., 2000; Riedemann et al., 2002a; Hotchkiss and Nicholson, 2006; Ward, 2008). Apoptosis appears to be the predominant factor that is responsible for lymphoid cell loss and the associated pathogenesis during sepsis (Song et al., 2000). It has been reported that early lymphocyte apoptosis in blood stimulated by sepsis in human being was associated with low survival rate (Le Tulzo et al., 2002). Apoptosis can be induced via both the extrinsic (TNF-α, Fas ligand) and intrinsic pathways (mitochondrial) during sepsis (Ward, 2010), and prevention of lymphoid cell apoptosis could markedly attenuate parameters of sepsis and improve survival (Hotchkiss et al., 2000; Oberholzer et al., 2001). *In vitro* experiments demonstrated that when exposed to C5a, thymocytes from septic rats showed increased apoptotic rate, which was attributable to the increased caspase-3, -6, and -9 activities (Riedemann et al., 2002a). However, C5a exposure alone could not stimulate normal thymocyte apoptosis (Guo et al., 2004), suggesting that other factors such as TNF-α and Fas ligand-induced by sepsis were indispensible for C5-indued apoptotic death of thymocytes. Furthermore, *in vivo* experimental data showed that thymocyte apoptosis was induced in a time-dependent fashion during sepsis, leading to around 50% loss of thymus weight 24 h after onset of sepsis (Guo et al., 2000). Thymocyte apoptosis was due to elevated ratio of apoptotic accelerators to anti-apoptotic proteins, because the activities of caspase-3, -6, -9 and cytochrome c-level in cytosol were significantly increased 12 h after CLP induction of sepsis, while Bcl-X_L_ content was greatly reduced (Guo et al., 2000). Importantly, C5a neutralizing antibody treatment maintained caspase-3, -6, and -9 activities at basal levels, prevented increase of ctyosolic cytochrome c concentration and decrease of Bcl-X_L_ level (Guo et al., 2000). These studies indicated that intrinsic pathway participated in sepsis-induced thymocyte apoptosis, which could be intervened by C5a blockade (Figure 2).

C5a can also contribute to apoptosis of other cell types (Figure 2). Recent study showed that C5a treatment caused significant apoptosis of adrenal medulla cells (PC12), leading to impaired generation of catecholamines in a dose- and time-dependent manner (Flierl et al., 2008a). *In vivo*, apoptosis of adrenal medulla cells was markedly increased after CLP-induced sepsis, which was greatly reversed by C5a blockade (Flierl et al., 2008a). Furthermore, pan-caspase inhibitor treatment prevented C5a-induced PC12 cell apoptosis during sepsis (Flierl et al., 2008a), suggesting that elevated caspase activities are critical for C5a-induced adrenal medulla cell apoptosis. Septic encephalopathy secondary to a breakdown of the blood-brain barrier (BBB) is a known complication of sepsis. Using CLP-induced sepsis model, a recent study demonstrated that the neutralization of C5a greatly ameliorated pathophysiological changes associated with septic encephalopathy (Flierl et al., 2009). Furthermore, C5a/C5aR signaling was also linked to increased caspase 3 activity and apoptosis in mouse brain endothelial cells (Jacob et al., 2011).

While C5a stimulated apoptosis of several cell types during sepsis, it provides anti-apoptotic signals to neutrophils (Figure 2). *In vitro* experiments showed that C5a inhibited spontaneous human neutrophil apoptosis by activating PI3-K/Akt signaling pathway (Perianayagam et al., 2002). In addition, C5a stimulation could lead to activation of ERK1/2 (Suvorova et al., 2008) and protein kinase C (PKC) (Simon, 2003). Both ERK1/2 and PKC can provide neutrophils with anti-apoptotic signals (Simon, 2003). Thus, C5a might be involved in delayed neutrophil apoptosis through multiple signaling pathways. It is noteworthy that C5a plays a key role in generation of inflammatory mediators such as IL-1β, IL-6, and IL-8 in humans (Strieter et al., 1992; Hopken et al., 1996), all of which can stimulate anti-apoptotic signals in neutrophils (Simon, 2003). We have previously observed that neutrophils from septic rats showed delayed spontaneous apoptosis when compared with those from normal animals (Guo et al., 2006). In contrast to normal serum, septic sera treatment led to significant resistance of neutrophils isolated from normal rats to apoptotic death, which was due to activation of both Akt and ERK1/2 (Guo et al., 2006). In sharp contrast, septic sera from rats receiving anti-C5a antibody restored the sensitivity of neutrophils to spontaneous apoptosis (Guo et al., 2006). C5a-induecd resistance of neutrophils to apoptosis was due to enhanced phosphorylation of Akt and ERK1/2, and increased expression of X-linked inhibitor of apoptosis and Bcl-X_L_ (Guo et al., 2006). These studies together suggest that the distinct effects of C5a on apoptosis in various cell types may induce different pathophysiology in sepsis. Increased apoptotic death of lymphocytes and adrenal medulla cells led to immunosuppression during sepsis, while decreased apoptotic rate caused release of more toxic cellular products from activated neutrophils (Figure 1). Together, these events may result in delayed pathogen elimination, normal tissue damages, and finally MOF.

## Effect of C5a on cardiac dysfunction during sepsis

**Figure 3:**
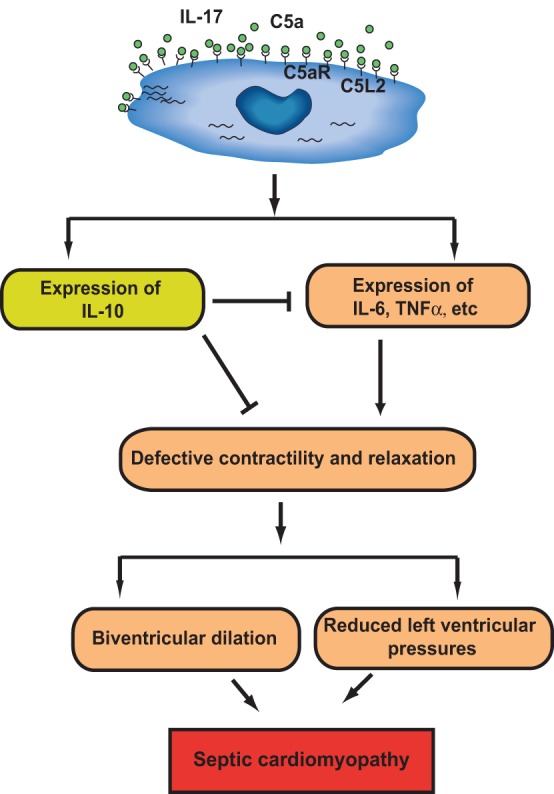
**Role of C5a signaling in cardiac dysfunction in sepsis.** In cecal ligation and puncture (CLP)-induced sepsis, expression of C5aR, but not C5L2, on CMs is increased. C5a increased production of inflammatory mediators (IL-6, TNF-α, etc.,) and anti-inflammatory mediator (IL-10) from CMs in a C5aR-, C5L2-, and IL-17-dependent mechanism. The resulting cytokine storm impaired CM contractility leading to “septic myocardiopathy.” C5a-dependent IL-10 may function as a negative feedback regulator of CM function during sepsis.

Defect in cardiac function is often induced in septic patients and has been referred to as “cardiomyopathy of sepsis.” “Septic cardiomyopathy” has been characterized by *in vitro* defective cardiomyocyte (CM) function. During sepsis, left ventricular pressures were greatly reduced, and CMs isolated from septic rats exhibited defective contractility and relaxation (Niederbichler et al., 2006). Importantly, when incubated with C5a, CMs isolated from both sham and CLP animals developed defective contractility and relaxation (Niederbichler et al., 2006). These defects were attenuated in septic rodents receiving anti-C5a antibody treatment, indicating that C5a might play a central role in cardiac dysfunction during sepsis. “Cardiosuppressive cytokines,” the definition of which is based on their ability to disrupt normal contractile function of normal CMs, have been described in patients with sepsis, and include IL-6, TNF-α, and IL-1β (Cain et al., 1999; Joulin et al., 2007; Ward, 2010). Furthermore, a recent study showed that polymicrobial sepsis greatly induced generation of inflammatory mediators in hearts, and CMs isolated from septic rodents spontaneously secreted cytokines and chemokines (IL-6, TNF-α, IL-1β, MIP-1α, MIP-2, MCP-1, KC, and IL-10) in a time-dependent manner (Atefi et al., 2011). In contrast, CMs obtained from septic rodents receiving neutralizing antibody to C5a produced significant less amount of the inflammatory mediators. Thus, C5a production during sepsis resulted in increased expressions of cytokines and chemokines in CMs, leading to cardiac dysfunction (Atefi et al., 2011) (Figure 3). The role of IL-10 in CM function during sepsis is unclear. IL-10 is considered to have anti-inflammatory effect and may be protective of septic heart by antagonizing other inflammatory mediators' effects, which represents a negative feedback mechanism regulated by C5a (Figure 3). In line with this hypothesis, a recent study shows that IL-10 prevents TNF-α induced cardiomyocyte apoptosis (Dhingra et al., 2011).

## Neutrophils and collateral tissue damage in sepsis

During CLP-induced sepsis, multiple organ failure occurs. When compared with control, CLP mice displayed higher level of plasma urea level, which indicated that the filtrating function of the kidneys was impaired (Dahlke et al., 2011). In addition, renal vascular permeability was significantly induced by septic shock, which was demonstrated by increased extravascular Evans blue leak (Benjamim et al., 2005). Liver cell damage and abnormal liver function were also induced by CLP as proved by elevated GOT/AST level—an indicator of liver cell injury, and bilirubin level, which suggested impairment of normal liver function (Dahlke et al., 2011). Moreover, CLP leads to pulmonary dysfunction. Histological assay showed that CLP-induced lung structural change such as alveolar swelling and inflammatory cell accumulation (Dahlke et al., 2011). Impairment of other organs, such as thymus, adrenal medulla, and heart, were also observed during septic shock (Riedemann et al., 2002a; Niederbichler et al., 2006; Flierl et al., 2008a). CLP-induced collateral tissue damages might be due to bacterial accumulation in lungs, kidneys, livers, and spleens (Riedemann et al., 2002b; Scott et al., 2003; Dahlke et al., 2011). However, whether CLP could induce bacterial burden in heart is still an open question. During sepsis-induced systemic inflammation, neutrophil influx into lungs and livers were elevated, as reflected by increased MPO activity in the corresponding organs (Scott et al., 2003; Dahlke et al., 2011). Transmigration of neutrophil from vascular vessels into collateral tissues is indispensible for bacterial clearance; however, excessive neutrophil accumulation could lead to tissue damages. It has been demonstrated that during CLP-induced sepsis, rat receiving anti-C5 antibody showed decreased bacterial load in spleen and liver compared with those receiving control IgG (Buras et al., 2004). In addition, anti-C5 treatment attenuated lung injury by reduced neutrophil influx (Buras et al., 2004), indicating that CLP might stimulate inappropriate neutrophil accumulation in tissues. Cardiac dysfunction-induced by CLP could be also alleviated by blocking C5a signaling (Niederbichler et al., 2006). Moreover, CLP-induced bacterial influx into lungs, kidneys, and livers could be reduced by disruption of C5aR (Riedemann et al., 2002b). Furthermore, disruption of C5aR could prevent thymocytes and adrenal medulla cells from apoptotic death (Riedemann et al., 2002a; Flierl et al., 2008a). However, the effect of C5aR on neutrophil accumulation in different organs and tissue (kidney, liver, lung and spleen) damages remains largely unknown. In addition, the role of C5L2 in bacterial dissemination, tissue accumulation of neutrophils, and organ damages is still enigmatic, though C5L2 deficient mice were resistant to CLP-induced systematic inflammatory reactions and subsequent death (Rittirsch et al., 2008).

## Expression and function of C5aR in sepsis

C5a can bind two receptors on the cells: C5aR and C5L2. C5aR (CD88) is a G-protein-coupled receptor with seven transmembrane segments. C5aR has a molecular weight of 45 kDa, and binds to C5a with high affinity, to a lesser extent, to C5a des Arg. The expression and function of C5aR in neutrophils during sepsis have been studied. After CLP in rats, C5aR content on neutrophils gradually decreased, reached the nadir at 24 h after onset of sepsis, and progressively increased thereafter (Guo et al., 2003). Mechanistically, the dynamic change of C5aR on neutrophil surface during sepsis might be due to internalization, followed by reconstitution (Guo et al., 2003). The result was consistent with the previous study that the association of C5a with C5aR caused rapid internalization of the ligand-receptor complex in neutrophils, followed by recycling of C5aR to the cell surface (Van Epps et al., 1990; Naik et al., 1997; Gilbert et al., 2001). Importantly, intravenous administration of neutralizing antibody to C5a markedly prevented decrease of C5aR content on neutrophils (Guo et al., 2003), suggesting that sepsis-induced rapid internalization of C5aR was likely caused by systemic appearance of C5a. Except for C5a-induced internalization, C5aR expression could also be regulated at transcription level by other inflammatory mediators generated during sepsis. For instance, C5aR mRNA expression was greatly reduced in monocytes and monocyte-derived dendritic cells by Th2 cytokine IL-4 (Soruri et al., 2003), which was significantly up-regulated during sepsis (Song et al., 2000). Surface content of C5aR on neutrophils might play an important role in their function. The lowest level of C5aR content on neutrophils 24 h after onset of CLP was accompanied by defective oxidative burst [decreased production of reactive oxygen species (ROS), especially H_2_O_2_] (Guo et al., 2003), which might be important for bacterial killing ability of neutrophils. Furthermore, the gradually increased expression of C5aR on neutrophils after 24 h CLP was correlated with elevated oxidative burst activity (Guo et al., 2003). Therefore, it seems that low level of C5aR on neutrophils might lead to reduced ROS production and followed high bacterial burden 24 h after CLP. However, the exact relationship between C5aR level and production of reactive nitrogen species (RNS) that may be more important for bactericidal activity is still unknown. On the other hand, there is no direct evidence demonstrating that reduced oxidative burst activity of neutrophils was due to decreased C5aR on surfaces; hence, use of C5aR knockout neutrophils is necessary to examine its influence on ROS and RNS expressions during CLP-induced sepsis.

The role of C5aR in sepsis was recently determined by gene knockout approach. In mid-grade CLP, 31% of wild type mice survived, whereas 80% of C5aR-deficient mice survived in a 7-days survival study, indicating the contribution of C5aR to harmful outcome of CLP-induced sepsis (Rittirsch et al., 2008). Furthermore, plasma levels of IL-1β, IL-6, MIP-2, and MIP-1α were obviously down-regulated in C5aR knockout mice when compared with wild type littermates (Rittirsch et al., 2008), suggesting that the harmful effects of C5aR during sepsis might result from C5a-mediated cytokine storm. Unfortunately, although C5aR blockade treatment resulted in lower bacterial burden in various organs, the influence of C5aR on bacterial counts was not investigated in C5aR-knockout mice. In line with this result, disruption of the C5a receptor gene significantly increases resistance to acute Gram-negative bacteremia, and endotoxic shock following an intravenous infusion of purified *E. coli* endotoxin (Hollmann et al., 2008). The role of C5aR in sepsis was also investigated by using a C5aR antagonist, C5aRa. C5aRa is a cyclic peptide to compete with C5a for binding to C5aR. During sepsis, C5aRa treatment blocked chemotactic responses of neutrophils to C5a, and prevented C5a/C5aR-induced paralysis of innate immunity, which led to improved survival in a 9-days survival study (Huber-Lang et al., 2002a). These studies further indicate C5aR as a potential therapeutic target in sepsis.

Originally, C5aR was thought to be exclusively expressed in myeloid cells such as macrophages, monocytes, neutrophils, basophils, and eosinophils (Solomkin et al., 1981; Gerard et al., 1989; Kurimoto et al., 1989; Werfel et al., 1992; Bosmann et al., 2012). There were now growing evidences that C5aR is expressed on a variety of non-myeloid cells. These include bronchial and alveolar epithelial cells, smooth muscle cells, Kupffer cells, endothelial cells, astrocytes, kidney tubular epithelial cells, and other parenchymal cells of solid organs such as lung, kidney, liver, and heart (Strunk et al., 1988; Gasque et al., 1995; Haviland et al., 1995; Lacy et al., 1995; Wetsel, 1995; Schieferdecker et al., 1997; Fayyazi et al., 2000; Zahedi et al., 2000; Drouin et al., 2001; Riedemann et al., 2002c; Sun et al., 2009). During the onset of experimental sepsis in rodents, up-regulated expression of C5aR was found in whole organs including lung, thymus, kidney, liver, and heart (Riedemann et al., 2002b) (Riedemann et al., 2003c), though CLP-induced C5aR level on neutrophils was reduced (Guo et al., 2003). Because lower C5aR level was accompanied by defective respiratory burst in neutrophils (Guo et al., 2003), disruption of C5aR function in other cell types except for neutrophils might contribute to improved survival rate during CLP-induced sepsis. Functionally, mice receiving blocking antibody to C5aR immediate after onset of CLP showed dramatically improved survival in a 7-days survival study (Riedemann et al., 2002b). Furthermore, anti-C5aR treatment led to a significant reduction of serum levels of IL-6 and TNF-α, and bacterial counts in a variety of organs (lung, liver, and kidney) when compared with normal IgG injection (Riedemann et al., 2002b). Using CLP-induced sepsis model in mice, IL-6 blockade was shown to have protective effects on sepsis, which are linked to reduced C5a receptor expression in lung, liver, kidney, and heart (Riedemann et al., 2003c). In another study, C5aR expression was markedly elevated on bronchial epithelial cells in LPS-induced systemic inflammation model (Drouin et al., 2001). However, the pathogenic role of C5aR signaling pathway in these organs during sepsis remains poorly understood.

C5aR was constitutively expressed in γδT cells and its expression was further enhanced in mice undergoing sepsis at both transcription and translation level (Han et al., 2011). *In vitro*, C5aR expression was elevated in γδT cells treated with C5a (Han et al., 2011), and incubation of γδT cells with C5a stimulated IL-17 expression (Han et al., 2011), implying the involvement of C5a/C5aR signaling in the release of inflammatory mediators from γδT cells during sepsis. Interestingly, our previous data showed that IL-17 released from γδT cells during experimental sepsis contributed to high concentrations of pro-inflammatory mediators and bacteremia, leading to a low survival rate (Flierl et al., 2008b).

C5aR expression in other cells and organs plays an important role in apoptosis during sepsis. C5aR expression was increased in thymocytes as early as 3 h after CLP, and peaked at 12 h (Riedemann et al., 2002a). The increased C5aR expression was accompanied by the elevated binding of C5a to the receptor on cell surfaces, leading to apoptosis-mediated loss of lymphoid cells (Riedemann et al., 2002a). Therefore, C5aR may be a possible therapeutic target to control unexpected apoptotic loss of lymphoid cells at the early stage of sepsis, preventing lethal immunosuppression. Clinically, catecholamines are frequently used last-resort drugs to prevent cardiovascular dysfunctions during severe sepsis. However, the mechanisms regulating their production during sepsis remain largely unknown. Recently, it was found that blockade of both C5aR and C5L2 abolished adrenomedullary apoptosis *in vivo* during sepsis, further suggesting that C5aR and C5L2 may be promising targets with implications on future complement-blocking strategies in the clinical setting of sepsis (Flierl et al., 2008a). C5aR in heart may also play a critical role in the development of reversible cardiac dysfunction commonly occurred during sepsis. A recent study demonstrated that C5aR mRNA level in hearts rose almost 3-fold as early as 6 h after CLP (Atefi et al., 2011). Furthermore, CMs isolated from C5aR- or C5L2-knockout rodents undergoing sepsis secreted low level of inflammatory mediators, which was comparable to sham group (Atefi et al., 2011).

C5aR was expressed in splenic NK and NKT cells (Fusakio et al., 2011). NK and NKT cells from C5aR knockout mice infected with *E. coli* expressed less CD69 (the marker of NK and NKT cell activation) when compared with their wild type counterparts, suggesting that C5aR signaling regulates the activation of NK and NKT cells (Fusakio et al., 2011). Furthermore, C5aR deficiency resulted in a reduced release of IFN-γ and TNF-α by NKT and NK cells and in an impaired recruitment of NKT and NK cells to the site of infection (Fusakio et al., 2011). Importantly, the absence of C5aR, NKT, and NK cells, but not of C5L2, led to significantly increased survival from sepsis, which was associated with reduced IFN-γ and TNF-α serum levels (Fusakio et al., 2011). These results together indicate that C5aR activation may represent a novel pathway driving detrimental effects of NKT and NK cells during sepsis. In addition, C5a and Toll-like receptor (TLR) acted synergistically to stimulate TNF-α and IFN-γ expressions in NK and NKT cells (Fusakio et al., 2011). Interestingly, the cognate antigen-mediated NKT cell activation was inhibited by C5a, suggesting that C5a might play a dual role in NKT cell activation (Fusakio et al., 2011).

## Role of C5L2 in sepsis

C5L2 is the newly identified C5a receptor, which has a molecular weight similar to C5aR. C5L2 belongs to a subfamily of C3a, C5a, and fMLP receptors, and like C5aR, it is expressed in various types of cell such as granulocytes and dendritic cells (Ohno et al., 2000). While C5L2 binds to C5a and C5a des Arg with high affinity, the interaction between C5L2 and other ligands such as C3a and C3a des Arg, is still a matter of controversy (Gerard et al., 2005; Kalant et al., 2005; Johswich et al., 2006; Chen et al., 2007; Scola et al., 2009). Unlike C5aR, C5L2 is uncoupled from G-proteins due to the replacement of arginine by leucine in the DRY region of the third intracellular loop, and the association of C5L2 with C5a induces no intracellular calcium influx (Okinaga et al., 2003; Scola et al., 2009). C5L2 was thus proposed to function as a recycling decoy receptor to remove active complement fragments from the extracellular environment (Scola et al., 2009). The majority of C5L2 are located in cytosol in the “resting” PMN, which was in striking contrast to C5aR that mainly appears to be on cell surfaces (Johswich et al., 2006; Scola et al., 2009). C5L2 can play both anti-inflammatory and pro-inflammatory roles. For example, C5L2 could protect mice from IgG immune complex-induced acute lung injury and inflammation (Gerard et al., 2005). Conversely, in a mouse model of OVA- or house dust mite-induced allergic asthma, C5L2 deficiency led to a attenuated asthmatic phenotype with the decreased airway hyper-responsiveness (AHR) and Th2 cytokine expression, and reduced airway accumulation of lymphocytes and eosinophils numbers as well as serum IgE level. Therefore, C5L2 may play opposite roles in distinct diseases (Zhang et al., 2010).

The functional role of C5L2 in sepsis remains poorly understood. In CLP-induced sepsis, C5L2 expression in neutrophils was increased, and C5L2 on cell surfaces did not undergo internalization as C5aR (Gao et al., 2005), suggesting the expression of C5aR and C5L2 are regulated by different mechanisms during sepsis. C5L2 expression was significantly increased in lung and liver in septic mice (Gao et al., 2005). Importantly, anti-C5L2 antibody-treated mice showed increased serum IL-6 level during CLP-induced sepsis (Gao et al., 2005). Furthermore, *in vitro* study using blood neutrophils showed that IL-6 expression-induced by LPS and C5a was further amplified by anti-C5L2 antibody treatment (Gao et al., 2005), indicating that C5L2 negatively regulated IL-6 generation. In line with these results, a recent study shows that TLR activation enhances C5a-induced pro-inflammatory responses in peripheral blood mononuclear cell (PBMC) and whole blood by negatively modulating the C5L2 (Raby et al., 2011). These data support the hypothesis that C5L2 could act as a “decoy” receptor to dampen inflammatory response during CLP-induced sepsis. Contrary to these speculations, C5L2 was shown to be a functional receptor rather than merely a decoy receptor (Rittirsch et al., 2008). In a mid-grade CLP model, 31% of wild type mice survived, whereas all C5L2 knockout mice survived in a 7-days survival study, suggesting a critical role of C5L2 in the harmful outcome of sepsis (Rittirsch et al., 2008). The effect of C5L2 during sepsis was linked to its regulation of both inflammatory cytokines (IL-1β, MIP-2, and MIP-1α) and plasma high mobility globulin β1 (HMGB1) in the blood (Rittirsch et al., 2008). These data suggest that C5L2 is a positive regulator of sepsis. In contrast to the finding in CLP model, C5L2-deficient mice showed increased susceptibility to lethal effects of LPS injection compared with control littermates (Chen et al., 2007). Furthermore, LPS-injected mutant mice showed higher IL-1β serum levels, indicating that the increased susceptibility was associated with elevation of some inflammatory cytokines (Chen et al., 2007). These results suggest that C5L2 plays a key role in the regulatory mechanism that protects against LPS-induced shock responses. Interestingly, C5L2 seems to have a functional role in heart during sepsis. Cardiomyocyte (CMs) isolated from wild mice undergoing sepsis produced high levels of IL-6, TNF-α, IL-1β, MIP-1α, MIP-2, MCP-1, and KC, while CMs from C5L2-deficient mice secreted significant low level of the inflammatory mediators (Atefi et al., 2011). These data suggest that C5aR and C5L2 contribute synergistically to the harmful consequences in heart during sepsis.

## Conclusions

Sepsis in human beings results in a high death rate. The therapeutic options remain limited and controversial. Following the recent updated review that no evidence suggests APC should be used for treating patients with severe sepsis or septic shock (Marti-Carvajal et al., 2012) and withdraw of Xigris [a recombinant human activated protein C (rhAPC)] from market in 2011, the search for “silver bullet” for the treatment of sepsis will continue. In septic human beings, there is abundant evidence for complement activation and C5a production. Interception of C5a or its receptors in the CLP model greatly improves survival in septic rodent. Mechanically, these observations are mainly linked to the recovery of blood neutrophil function during sepsis. Thus, anti-C5a strategy holds great promise for the treatment of sepsis. Eculizumab (trade name Soliris), a recombinant humanized monoclonal antibody that inhibits C5 cleavage by the C5 convertase via binding to C5 was recently approved for atypical hemolytic-uremic syndrome (aHUS), a disease that causes abnormal blood clots to form in the kidneys (2011). This will encourage the development of effective humanized monoclonal antibody targeting C5a or its receptors.

On the other hand, the molecular signaling whereby C5a/C5aRs regulates neutrophil function at different stages of sepsis remains poorly understood. Furthermore, although both C5aR and C5L2 are expressed in various other cell types and organs, their potential role in organ function during sepsis are not known. Importantly, many anti-C5a antibodies also bind C5, thus preventing the formation of the terminal complement complex C5b-9 which is important for controlling bacterial infection. Clearly, the anti-C5a strategy remains to be carefully evaluated in future clinical research and trials. Interestingly, a recent study shows that resolvin 2 (RvD2), a new member of lipid mediators enzymatically generated within resolution networks that possess unique and specific functions to orchestrate catabasis, potently reduced C5a-mediated neutrophil-endothelial interactions to reduce microbial peritonitis (Spite et al., 2009). Furthermore, RvD2 significantly inhibited C5a-stimulated extracellular superoxide generation (Spite et al., 2009). In CLP-induced sepsis, RvD2 sharply decreased the excessive cytokine production, neutrophil recruitment, bacterial burden while increasing peritoneal mononuclear cells and macrophage phagocytosis (Spite et al., 2009). These pro-resolving actions together translate to increased survival from CLP-induced sepsis (Spite et al., 2009). It is tempting to speculate that C5a/C5aRs signaling pathway may be a major target of resolvins. Understanding how the mechanisms by which activation of C5a/C5aR/C5L2 regulate cell and organ function including inflammatory responses and apoptosis is no doubt a fruitful field for future progress in prevention and treatment of sepsis.

### Conflict of interest statement

The authors declare that the research was conducted in the absence of any commercial or financial relationships that could be construed as a potential conflict of interest.
